# Pathophysiological Mechanisms of Sino-Atrial Dysfunction and Ventricular Conduction Disease Associated with *SCN5A* Deficiency: Insights from Mouse Models

**DOI:** 10.3389/fphys.2012.00234

**Published:** 2012-07-06

**Authors:** Christopher L.-H. Huang, Lily Lei, Gareth D. K. Matthews, Yanmin Zhang, Ming Lei

**Affiliations:** ^1^Physiological Laboratory, Department of Biochemistry, University of CambridgeCambridge, UK; ^2^Murray Edwards College, University of CambridgeCambridge, UK; ^3^Institute of Cardiovascular Sciences, University of ManchesterManchester, UK

**Keywords:** *SCN5A*, progressive cardiac conduction disease, sinus node dysfunction, mouse genetic models

## Abstract

Genetically modified mice provide a number of models for studying cardiac channelopathies related to cardiac Na^+^ channel (*SCN5A)* abnormalities. We review key pathophysiological features in these murine models that may underlie clinical features observed in sinus node dysfunction and progressive cardiac conduction disease, thereby providing insights into their pathophysiological mechanisms. We describe loss of Na^+^ channel function and fibrotic changes associated with both loss and gain-of-function Na^+^ channel mutations. Recent reports further relate the progressive fibrotic changes to upregulation of TGF-β1 production and the transcription factors, *Atf3*, a stress-inducible gene, and *Egr1*, to the presence of heterozygous *Scn5a* gene deletion. Both changes are thus directly implicated in the clinically observed disruptions in sino-atrial node pacemaker function, and sino-atrial and ventricular conduction, and their progression with age. Murine systems with genetic modifications in *Scn5a* thus prove a useful tool to address questions concerning roles of genetic and environmental modifiers on human *SCN5A* disease phenotypes.

## Introduction

The *SCN5A* gene directs synthesis of cardiac-type, voltage-dependent, Na^+^ channels (Na_v_1.5), abundant in the heart. The simultaneous and transient openings of large numbers of voltage-gated Na^+^ channels mediate the rapid inward current responsible for the rising phase of the cardiac action potential (AP). This is fundamental to initiation, propagation, and maintenance of the normal cardiac rhythm that ensures synchronized atrial and ventricular contraction and therefore the normal heartbeat. *SCN5A* mutations are associated with cardiac arrhythmic syndromes ranging from chronic bradyarrhythmias to acute life-threatening tachyarrhythmias. These include the congenital long QT syndrome subtype 3 (LQT3; Wang et al., 1995), Brugada syndrome (BrS; Chen et al., 1998; Brugada et al., 2000), isolated cardiac conduction disease (CCD; Schott et al., 1999), sinus node dysfunction (SND; Benson et al., 2003), and sudden infant death syndrome (SIDS; Otagiri et al., 2008). Over the past decade, mouse models with genetic modifications in *SCN5A* have provided invaluable insights into these cardiac disorders. This review summarizes recent studies on Na_v_1.5 disorders and their bearing on cardiac conduction properties.

## The Role of Voltage-Gated Na^+^ Channels in Cardiac Pacemaker Function

The last two decades have seen significant progress in our understanding of the molecular structure of voltage-gated Na^+^ channels (reviews Goldin, 2001, 2002; Catterall et al., 2003). These channels comprise pore-forming α-subunits, with molecular weights of ~260 kDa, and associated auxiliary β-subunits with molecular weights ~36 kDa (Goldin, 2002; Catterall et al., 2003). Expression of the α-subunit alone is sufficient for functional Na^+^ current expression, but the presence or absence of β-subunits modifies the kinetics and voltage-dependence of channel gating. The α-subunits are organized into four homologous domains (I–IV). Each contains six transmembrane α-helices (S1–S6) and includes an additional pore loop located between the S5 and S6 helices. The various α-subunit isoforms are differentially expressed in different tissues and have distinct pharmacological properties (Goldin, 2002; Catterall et al., 2003).

Recent investigations also demonstrate that in addition to Na_v_1.5, different cardiac tissues co-express a range of different α-subunit isoforms. These include several neuronal, Na_v_1.1, Na_v_1.3, and Na_v_1.6, isoforms primarily expressed in brain (Maier et al., 2002; Lei et al., 2004). This pattern is exemplified by the SA node, which shows a complex co-expression of multiple Na_v_ isoforms. It contains both Na_v_1.5 and Na_v_1.1 with Na_v_1.5 absent in the central nodal cells (Lei et al., 2004). Nav1.3 is also present in mouse (Maier et al., 2002) but not rabbit or rat SA node (Baruscotti et al., 1997; Maier et al., 2002). More recent, competitive RT-PCR and immunohistological, studies have demonstrated Nav1.1, Nav1.2, and Nav1.5 expression in canine SA and atrioventricular (AV) nodes (Haufe et al., 2005).

Recent studies have also clarified the functional roles of these distinct cardiac and neuronal-type Na^+^ channels in the SA node. First, it is likely that the tetrodotoxin (TTX)-sensitive neuronal (Na_v_1.1) Na^+^ current is involved in pacemaker function in the SA node. Nanomolar TTX concentrations known to only inhibit neuronal Na^+^ current slowed down pacemaker rates of intact mouse hearts by ~65% (Maier et al., 2002) isolated SA nodes by ~22% and isolated SA nodal pacemaker cells by ~15% (Lei et al., 2004). Furthermore, studies using the AP clamp technique demonstrated that the neuronal Na^+^ current could be activated within the voltage ranges of the pacemaker potential (Lei et al., 2004).

In contrast, the TTX-resistant cardiac (Na_v_1.5) Na^+^ current is likely to be important in AP propagation and conduction through the SA node. Thus, measurements of SA node conduction times demonstrated that block of both TTX-sensitive and TTX-resistant Na^+^ current by μM TTX slowed or even blocked AP conduction through the SA node periphery, from the leading pacemaker site in the center of the SA node to the surrounding atrial muscle. However, block of the TTX-sensitive Na^+^ current alone by 10 or 100 nM TTX did not produce such effects (Lei et al., 2004).

Together these findings implicate both neuronal (Na_v_1.1) and cardiac (Na_v_1.5) Na^+^ channels in pacemaker activities as well as AP conduction through the SA node and from the SA node to surrounding atrial muscle. Their demonstration of physiological roles for Na_v_1.5 in SA node function underpins our understanding of the SND attributed to genetic defects in Na_v_1.5 channel, as discussed in the sections below.

## *SCN5A* Mutations and Human Cardiac Conduction Diseases

### Sinus node dysfunction

Sinus node dysfunction is associated with abnormal impulse formation and propagation in the SA node. It presents clinically as sinus bradycardia, sinus pause or arrest, atrial chronotropic incompetence, and SA node exit block (Freedman, 2001). It affects ~1 in 600 cardiac patients older than 65 years and is responsible for ~50% of the million permanent pacemaker implants per year worldwide (de Marneffe et al., 1993; Dobrzynski et al., 2007). Thus far, 13 human Nav1.5 (hNav1.5) mutants have been associated with familial SND (reviews: Lei et al., 2007, 2008). In expressed heterologous systems, these fall into three groups respectively generating peak Na^+^ currents that were comparable to those observed for wild-type hNav1.5 (e.g., L212P, P1298L, DelF1617, and R1632H), significantly reduced but nevertheless detectable (e.g., E161K, T220I, and D1275N) or undetectable (e.g., T187I, R878C, G1408R, and the truncated variants W1421X, K1578fs/52, and R1623X; Gui et al., 2010). Their comparisons with the corresponding clinical outcomes suggest that *SCN5A* haploinsufficiency markedly reducing Na^+^ current can result in SND. However, disease phenotypes can be accompanied by other cardiac excitation disorders: further factors besides Na^+^ current reduction and aging may also contribute to development of SND phenotypes (Leoni et al., 2010).

### Progressive cardiac conduction disease

Progressive cardiac conduction disease (PCCD), also known as Lev–Lenegre disease (Schott et al., 1999), presents as a progressive prolongation of electrocardiographic cardiac conduction parameters. PCCD is most commonly seen in the elderly, and has been attributed to senile degeneration of the conduction system. Hereditary PCCD has been attributed to loss-of-function *SCN5A* mutations that reduce Na^+^ current by decreasing sarcolemmal expression of channel proteins, causing an expression of non-functional channels, or altering channel gating properties through delayed activation, earlier inactivation, enhanced slow inactivation, or slowed recovery from inactivation (Tan et al., 2001; Probst et al., 2003; Zimmer and Surber, 2008). Moreover, a single *SCN5A* mutation may cause either an isolated PCCD or an overlap syndrome with PCCD co-existing with BrS (Zimmer and Surber, 2008). This suggests that in addition to reductions in Na^+^ current sufficient to slow conduction in PCCD or BrS, other factors contribute to the development of the typical right-precordial ST segment elevation in BrS. Further reports describe overlaps between gain- and loss-of-function phenotypes associated with a single C-terminal 1795insD gene mutation yielding ECG features of bradycardia, conduction disease, LQT3, and BrS (Bezzina et al., 1999).

## Mouse Models of Cardiac Conduction Diseases Associated with *SCN5A* Mutations

A mouse model containing a disrupted Na_v_1.5 gene, *Scn5a*, was developed a decade ago (Papadatos et al., 2002). Homozygotes show intrauterine lethality with severe defects in ventricular morphogenesis. *Scn5a*^+/−^ heterozygotes show normal survival but a number of electrophysiological defects including impaired atrioventricular conduction, delayed intramyocardial conduction, increased ventricular refractoriness, and ventricular tachycardia with characteristics of re-entrant excitation. Single whole-cell patch clamp studies of isolated adult *Scn5a*^+/−^ ventricular myocytes demonstrated a ~50% reduction in Na^+^ conductance. Such mice thus provide a unique and valuable model system for studying physiological effects of cardiac Na^+^ channelopathies.

### Loss of Na_v_1.5 channel function and sinus node dysfunction

**Figure 1:**
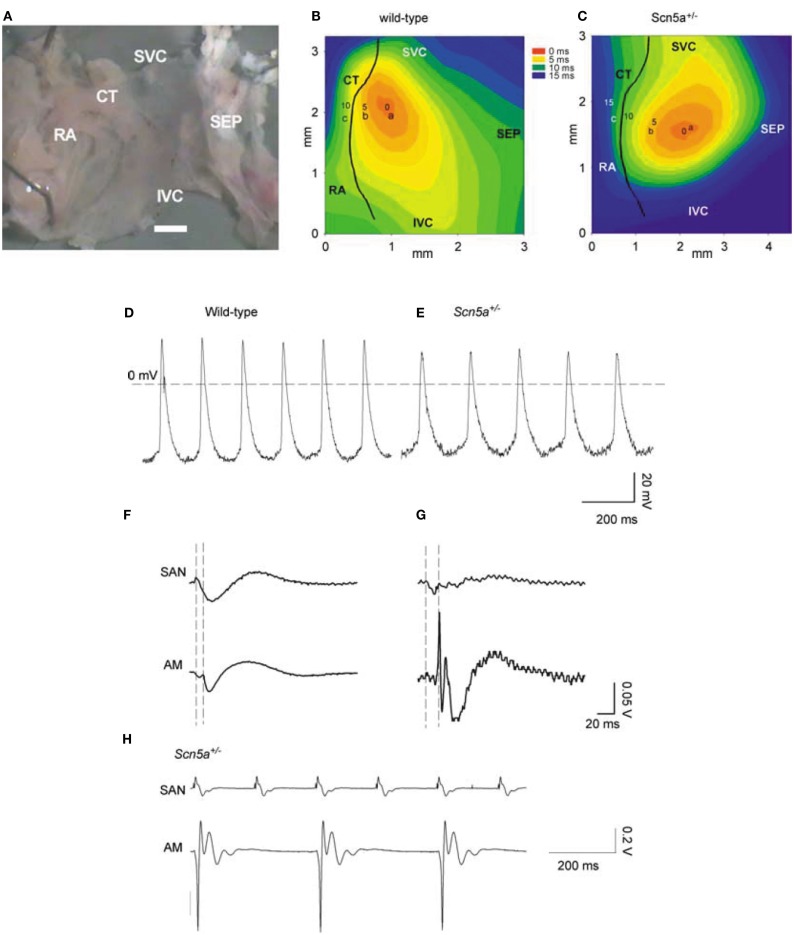
**SAN pacemaking and conduction in WT and *Scn5a*^+/−^ mice**. **(A)** Example of SAN preparation used for electrical mapping. Scale bar, 200 μm. **(B,C)** Activation sequence in SAN of WT and *Scn5a*^+/−^. **(D,E)** Action potentials recorded from sites near the center of the SAN in **(A,B)** from WT (left) and *Scn5a*^+/−^ (right) SAN preparations. **(F,G)** SAN conduction in WT and *Scn5a*^+/−^ preparations. Extracellular potentials from sites a [leading pacemaker site in the center of the SAN; see **(B,C)**] and c (atrial muscle, AM) shown. Vertical dashed lines indicate the time of initiation of the AP at the leading pacemaker site (left) and the arrival of the AP in atrial muscle (right). **(H)** Simultaneous SAN and atrial muscle recordings showing sino-atrial conduction block in *Scn5a*^+/−^ SAN, never observed in WT. SEP, septum; SVC, superior vena cava; IVC, inferior vena cava; CT, crista terminalis; and RA, right atrial appendage.

Long-term telemetric ECG recordings demonstrated that *Scn5a*^+/−^ mice retain physiological circadian variations in heart rates but replicated the depressed mean rates and persistent SA block observed in patients with SND (Asseman et al., 1983). Isolated hearts similarly showed a sinus bradycardia, slowed SA conduction, and sino-atrial exit block. Isolated *Scn5a*^+/−^ SA node and atrial preparations (Figure 1) showed slowed SA conduction and frequent SA conduction block. Patch clamp analyses of *Scn5a*^+/−^ SA node cells demonstrated similar steady-state activation and inactivation properties but reduced maximum Na^+^ currents (~30%) compared to WT (Lei et al., 2005). These studies together implicate the *Scn5a* Na^+^ channel in both pacemaker and conduction functions of the mouse SA node, and in the clinical consequences of SND.

### Loss of Na_v_1.5 channel function and progressive cardiac conduction disease

*Scn5a*^+/−^ mice have been further investigated for potential pathophysiological mechanisms involved in the progressive evolution of *SCN5A*-related PCCD (Royer et al., 2005; van Veen et al., 2005). Atrial, atrioventricular, and ventricular conduction velocities were all prolonged to extents that increased with age. This feature was confirmed by activation mapping studies performed in Langendorff-perfused hearts. In young *Scn5a*^+/−^ mice, conduction velocity was only affected in the right ventricle. In old mice, the right ventricular conduction defect worsened and was also associated with conduction velocity defects in the left ventricle. This age-dependent deterioration of ventricular conduction was associated with the occurrence of fibrosis in the ventricular myocardium (Jeevaratnam et al., 2011).

### Gain of Na_v_1.5 function and sinus node dysfunction

SA node function in mice heterozygous for a knock-in, gain-of-function KPQ-deletion (*Scn5a*^+/Δ^) also appears to recapitulate major features of SND reported in LQT3 patients, suggesting phenotypic overlaps with *loss*-of-function Na^+^ channel mutations. Abrupt accelerations in heart rate or premature beats thus caused AP lengthening, early after depolarizations, and triggered arrhythmias (Nuyens et al., 2001). Electrophysiological characterizations of SA node function in intact *Scn5a*^+*/*Δ^ mice and *in vitro* sino-atrial preparations were compared with features of cellular SA node and two-dimensional tissue models exploring consequences of *Scn5a*^+*/*Δ^ mutations. Whilst showing prolonged electrocardiographic QT and QTc intervals expected for LQT3, *Scn5a*^+*/*Δ^ mice showed frequent episodes of sinus bradycardia, sinus pause/arrest, and increased sinus node recovery times, suggesting compromised pacemaker activity, as well as depressed intra-atrial, atrioventricular node, and intraventricular conduction. Isolated SA preparations correspondingly showed lower mean intrinsic heart rates and slower conduction both within the SA node and from SA node to surrounding atrium. Modeling studies reconstructed such findings through a combination of augmented tail and reduced peak Na^+^ currents attributable to downregulation of a Na^+^ channel that itself shows increased tail currents as a result of a gain-of-function mutation (Wu et al., 2012).

Veldkamp et al. (2003) also studied the effects of the 1795insD Na^+^ channel mutation on SA pacemaker function using experimental models in HEK-293 cells and computer simulation. They demonstrated late Na^+^ currents from the 1795insD channels whose magnitude varied between 0.8 ± 0.2 and 1.9 ± 0.8% of peak Na^+^ current. AP clamp experiments confirmed an existence of 1795insD channel activity during SA node pacemaker function. Computational models for the SA node AP then incorporated late Na^+^ current and the negative shift in voltage-dependence of inactivation. Such negative shifts decreased the sinus rate by decreasing the diastolic depolarization rate, whereas the presence of late Na^+^ current decreased the sinus rate by AP prolongation, despite a concomitant increase in diastolic depolarization rate. This suggested that Na^+^ channel mutations displaying a late Na^+^ current or a negative shift in inactivation may account for the bradycardia seen in LQT3 patients, whereas SA node pauses or arrest may result from failure of SA node cells to repolarize under conditions of extra net inward current (Veldkamp et al., 2003).

### Gain of Na_v_1.5 function and conduction failure

The overlap patterns indicated above involving gain- and loss-of-function phenotypes can also involve atrial and ventricular conduction. 1795insD hearts combine a preferential right ventricular conduction slowing accompanied by reduced peak sodium current densities, and AP upstroke velocities with AP prolongation, slowed Na^+^ current decays but normal activation and inactivation characteristics, and increased persistent inward currents compared to WT (Remme et al., 2006). Increased atrial arrhythmic tendencies in aging gain-of-function *Scn5a*^+/Δ^ are accompanied by reductions in conduction velocity that could result from downregulation of Nav1.5 in contrast to its increased expression in WT hearts (Guzadhur et al., 2010).

## Recent New Insights into the Molecular Basis of *SCN5A* Mutations and Human Cardiac Conduction Diseases

**Figure 2:**
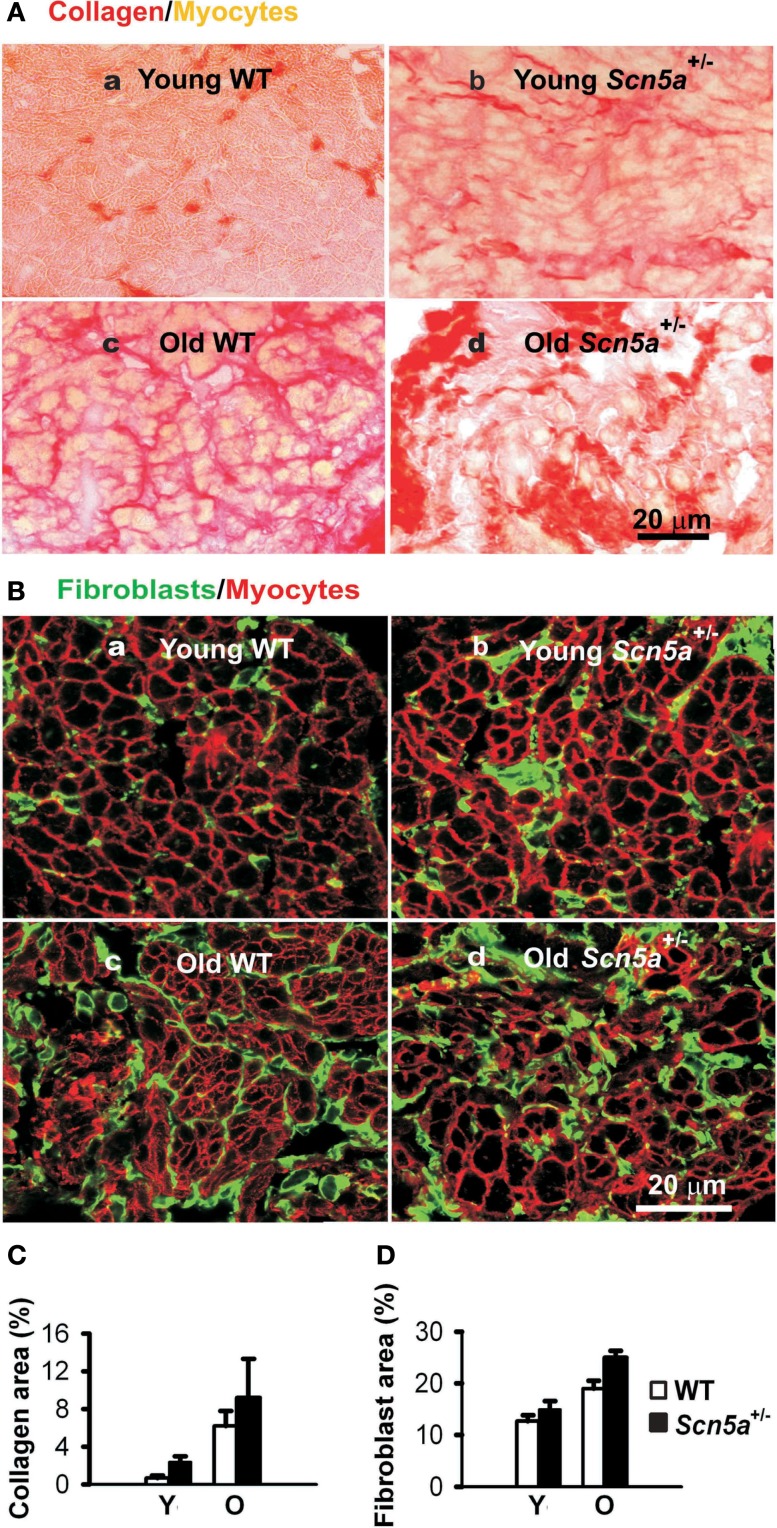
**Characterization of remodeling of extracellular matrix in the SAN**. **(A)** Picosirus red collagen staining in SAN section from each of four groups of mice. **(B)** Vimentin immunostaining for fibroblasts in SAN tissue sections. **(C)** Collage quantification: area of picosirus red-stained tissue expressed as a percentage of the field of view. **(D)** Fibroblast quantification with area of vimentin staining expressed as a percentage of the field of view. Y, young mice; O, old mice. Two-way ANOVA: *P *< 0.05, *Scn5a*^+/−^ vs. WT mice; *P *< 0.001, young vs. old mice.

Recent studies have explored for possible relationships between the pathogenesis of SND, aging processes, and *Scn5a-*disruption, as well as their possible interactions in the *Scn5a*^+/−^ mouse model (Hao et al., 2011). These associated both electrical remodeling and tissue degeneration, detected as a TGF-β_1_-mediated fibrosis, with altered pacemaker and conduction function in SND. Such changes occurred with both *Scn5a*-disruption and aging. A combination of both factors produced the most severe phenotype. Thus, *ex vivo* SA node preparations isolated from their autonomic inputs then showed increased cycle lengths and sino-atrial conduction times. These changes accompanied alterations in the extent of fibrosis assessed by collagen and fibroblast levels, and ion channel and regulatory gene transcriptional remodeling. Aging and *Scn5a*-disruption correspondingly resulted in interacting up-regulatory effects on levels of the key modulator of fibrosis, TGF-β_1_, and the fibroblast marker, vimentin. The latter changes were also greatest in the old *Scn5a*^+/−^ hearts (Figure 2). Altered expression of TGF-β_1_ and vimentin transcripts are associated with increased collagen and fibroblast abundance indicating an occurrence of interstitial fibrosis. The fibrosis could potentially slow conduction both within the SA node and from the SA node to the surrounding atrium. Its occurrence parallels previous reports associating the *Scn5a*^+/−^ condition and similar, ventricular, fibrotic changes (Royer et al., 2005; van Veen et al., 2005) as well as associating age-dependent SND and fibrosis (Benditt et al., 1990) although such features were not observed in all studies (Alings et al., 1990; Alings and Bouman, 1993). In implicating Na_v_1.5 deficiency in such changes these findings additionally suggest novel regulatory roles for Na_v_1.5 in cellular biological processes extending beyond its electrical function.

The above findings add to studies that similarly showed that heterozygous *Scn5a* inactivation in mouse produces ventricular rearrangements and fibrosis with aging (Royer et al., 2005; van Veen et al., 2005; Leoni et al., 2010). They also demonstrated aging resulted in an upregulation of two transcription factors, *Atf3*, a stress-inducible gene, and *Egr1*, an early growth response gene, particularly in *Scn5a*^+/−^ mice (Royer et al., 2005; van Veen et al., 2005). Furthermore, the variable Na_v_1.5 protein expression from the WT allele correlates with the extent of PCCD in the *Scn5a*^+/−^ mouse model (Leoni et al., 2010). This study (Leoni et al., 2010) divided 10-week-old *Scn5a*^+/−^ mice into two electrocardiographic subgroups showing either severe or mild ventricular conduction defects. These phenotypic differences persisted with aging. At 10 weeks, the Na^+^ channel blocker ajmaline produced similar prolongations of QRS intervals in both groups of *Scn5a*^+/−^ mice. However, the effect of ajmaline was greater in the severely affected subgroup in old mice (>53 weeks). Ventricular tachycardia developed in 5- to 10-week-old severely but not mildly affected *Scn5a*^+/−^ mice. Such findings therefore matched clinical observations in patients with *SCN5A* loss-of-function mutations with either severe or mild conduction defects. The severely but not mildly affected old *Scn5a*^+/−^ mice also showed extensive cardiac fibrosis. Finally, the severely affected *Scn5a*^+/−^ mice had similar Na_v_1.5 mRNA but lower Na_v_1.5 protein expression, moderately smaller Na^+^ currents, and reduced AP upstroke velocities than the mildly affected *Scn5a*^+/−^ mice.

## Conclusion

Mice have become powerful tools for clarifying the pathophysiological consequences of *SCN5A* mutations in *SCN5A*-related cardiac disorders. Mice harboring *SCN5A* mutations related to PCCD and SND convincingly recapitulate the clinical phenotypes of PCCD and SND in patients, and provide insight into the mechanisms of *SCN5A* deficiency-associated cardiac conduction diseases. They should constitute useful tools for future studies addressing as yet unanswered questions, such as the role of genetic and environmental modifiers on *SCN5A* disease phenotypes.

## Conflict of Interest Statement

The authors declare that the research was conducted in the absence of any commercial or financial relationships that could be construed as a potential conflict of interest.
